# Italian weedy rice—A case of de‐domestication?

**DOI:** 10.1002/ece3.6551

**Published:** 2020-07-12

**Authors:** Annabelle Grimm, Vaidurya P. Sahi, Manuel Amann, Francesco Vidotto, Silvia Fogliatto, Katrien M. Devos, Aldo Ferrero, Peter Nick

**Affiliations:** ^1^ Molecular Cell Biology Botanical Institute Karlsruhe Institute of Technology Karlsruhe Germany; ^2^ Dipartimento di Scienze Agrarie Forestali e Alimentari Universita degli Studi di Torino Torino Italy; ^3^ Institute of Plant Breeding, Genetics and Genomics (Department of Crop and Soil Sciences), and Department of Plant Biology The University of Georgia Athens GA USA

**Keywords:** domestication, endoferality, exoferality, single nucleotide polymorphisms, weedy rice (*Oryza sativa cf. spontanea*)

## Abstract

Weedy rice is a representative of the extensive group of feral weeds that derive from crops, but has returned to the lifestyle of a wild species. These weeds develop either from a hybridization of crops with wild relatives (exoferality), or by mutation of crops to weedy forms (endoferality). Due to the close relation of weed and crop, the methods for weed‐targeted containment are limited to date. A deeper understanding of the development of such weeds might help to design more efficient and sustainable approaches for weed management. Weedy rice poses a serious threat to rice yields worldwide. It is widely accepted that weedy rice has originated independently in different regions all over the world. However, details of its evolution have remained elusive. In the current study, we investigated the history of weedy rice in northern Italy, the most important rice‐growing area in Europe. Our approach was to analyze genes related to weedy traits (*SD1, sh4, Rc*) in weedy rice accessions compared to cultivars, and to integrate these results with phenotypic and physiological data, as well as historical information about rice farming in Italy. We arrive at a working model for the timeline of evolution of weedy rice in Italy indicating that both exoferality and endoferality acted as forces driving the development of the diverse weedy rice populations found in the region today. Models of weed evolution can help to predict the direction which weed development might take and to develop new, sustainable methods to control feral weeds.

## Graphical abstract

Using genes for domestication traits, the evolution of Weedy Red Rice in Italy is shown to be composed of exoferality (introgression from alleles originating from wild rice, transmitted by Indian landraces) and endoferality (mutation of domestication alleles).

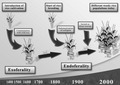

## 1 INTRODUCTION

Weeds and invasive plant species impair agriculture worldwide. They coexist with crops in agricultural habitats and often are close relatives of crops. However, crops and weeds are subject to different selection pressures, and therefore, weeds evolve into different, undesired directions (Ellstrand et al., 2010). Through competition with crop plants, weeds reduce crop yields substantially and are therefore considered highly problematic.

Two concurrent models have been proposed for weed evolution, and both are supported by experimental evidence. One scenario assumes that invasion by a crop wild relative is followed by hybridization with the respective crop, resulting in feral forms that spread over the agricultural area. This process is referred to as exoferality. The other model assumes that weeds might originate from the crop itself by spontaneous mutations that predispose the plant to adapt to a different set of selection pressures channeling the subsequent evolution toward a “weedy lifestyle.” This pathway of weed development is called endoferality. These processes and underlying mechanisms have been discussed in detail by Ellstrand et al. (2010). Meanwhile, it is widely accepted that the evolution of crop weeds is driven by human activity (Dekker, 2011), since agriculture administers selection pressures that favor the evolution of weedy traits.

This blurred borderline between crop and weed leads to a couple of terminological difficulties. *Cultivars* are genetically homogenous cultivated varieties that have been registered by seed authorities. In contrast, *landraces* or old varieties usually do not meet this criterion and should be distinguished as *cultigens*. By the term *weedy accession*, we describe individuals recovered from agricultural sites, but displaying de‐domestication traits, while *wild genotypes* have never been domesticated. This nomenclature will be used throughout the current study.

Weedy rice, the dominant weed of rice paddies, occurs in rice‐growing areas all over the world and causes severe yield losses.

Today, it is generally accepted that weedy rice has originated independently in different geographical areas of the world. Both exoferality and endoferality have been shown to contribute to the establishment of weedy rice populations (De Leon et al., 2019; Huang et al., 2017; Li, Li, Jia, Caicedo, & Olsen, 2017; Reagon et al., 2010; Song, Chuah, Tam, & Olsen, 2014; Vigueira et al., 2019). However, the specific mechanisms underlying the development of weedy rice in the respective geographical areas have largely remained unsolved. This is not a question of mere academic interest, but of eminent importance for agronomy: To date, no gold standard for the management of weedy rice could be established. Even the success of herbicide‐resistant cultivars is limited to a few seasons, due to gene flow that can transfer the resistance to weedy rice (Burgos et al., 2014; Wang et al., 2013). Therefore, it is fundamental to understand the forces driving its evolution, in order to develop containment strategies that are both reliable and sustainable.

Weedy rice is characterized by a set of attributes such as increased plant height, severe seed shattering, and a red pericarp to just name the most obvious examples (Fogliatto, Vidotto, & Ferrero, 2012). It is these traits that cause the massive problems following infestation with weedy rice: This weed easily outcompetes the cultigens, causing severe yield losses. Moreover, the seeds enter the seed bank after shattering and, due to their dormancy, remain viable for years (Gu et al., 2011). This makes it extremely difficult to cure infested soils.

Reducing plant height has been a key target for cereal breeding, because it is directly correlated with lodging resistance. Most of the recent rice cultivars are so called semi dwarf varieties, optimized for stunted culms that are able to support panicles of higher weight, thus granting increased yields. The trend for reduced stature started in the 1960s and was one of the most important drivers for the Green Revolution (Khush, 1999). The genetic target which has been modified by the breeding of semi dwarf varieties, the locus *semi dwarf 1* (*SD1*), is located on rice chromosome 1 and encodes the gibberellin oxidase GA2OX2 (Jia et al., 2019; Sasaki et al., 2002; Spielmeyer, Ellis, & Chandler, 2002).

Shattering is considered one of the most problematic traits of weedy rice since it leads to the formation of a seed bank in the soil. The *SH4* locus, previously referred to as *SHA1* (for shattering), located on chromosome 4 plays a key role in shattering in rice. A transversion from G to T at position 237 in exon 1 of *SH4* has been linked with a dramatic reduction of shattering in domesticated rice (Li, Zhou, & Sang, 2006). However, this has been challenged by several studies which reported weedy rice accessions that carry said mutation but still show severe seed shattering (Subudhi et al., 2014; Thurber et al., 2010; Zhu, Ellstrand, & Lu, 2012). The *SH4* locus encodes a myb3 transcription factor (Li et al., 2006), and is one of the most thoroughly studied genes associated with shattering.

Weedy rice sometimes is also referred to as red rice, due to the reddish color of the caryopses, resembling that of wild rice species. Pericarp pigmentation is under control of *Rc*, a bHLH transcriptional regulator of proanthocyanidin synthesis, located on chromosome 7. A 14‐bp deletion in exon 7 of the *Rc* gene played a major role in domestication, because it led to white varieties of rice (Gu et al., 2011; Sweeney et al., 2007). The deletion leads to a frame shift resulting in a premature stop codon that inactivates the DNA‐binding domain, rendering the transcription factor nonfunctional (Furukawa et al., 2006). The functional Rc protein in wild rice is tightly associated with seed dormancy and longevity (Pipatpongpinyo et al., 2020).

Italy, as the main producer of rice in Europe, suffers from severe infestations of weedy rice. In addition, due to its relatively young and well‐documented history of rice agriculture, and the absence of autochthonous crop wild relatives, Italy represents a perfect model area to follow the history of weedy rice along with the cultivated varieties in the region, and to gain an understanding of the evolution of weeds within agro‐ecosystems. During an initial study (Grimm, Fogliatto, Nick, Ferrero, & Vidotto, 2013), we assessed genetic diversity in Italian populations of weedy rice along with sympatric cultivated varieties with a SSR marker set from Cao et al. (2006). Most strikingly, some of the cultivated varieties were found to cluster with, weedy rice. With only one exception, these cultivated varieties were old landraces originating from the 19th century that mostly had been abandoned more than a century ago. Because of their genetic proximity to weedy rice, and their higher variability, these landraces were seen as potential ancestors of the weedy rice populations in Italy. However, since the study was based on neutral SSR markers, it was not possible to draw conclusions on the mechanisms driving the evolution of weedy rice in the Piemonte region.

The current study was initiated by the null hypothesis that weedy alleles of domestication genes might have supported the introgression of weedy traits into these historic landraces. An implication of this hypothesis would be that we should be able to detect, for genes for domestication‐related traits, either alleles also found in the wild ancestors of *O. sativa*, or mutated versions of the domesticated allele that have regained the functionality they had in the wild rice ancestor. In fact, the current study shows that both implications can be confirmed. This allowed to gain insight into the evolution of weedy rice in the sampling area, leading to an extended working model on the timeline of weedy rice evolution in northern Italy.

## 2 MATERIAL AND METHODS

### 2.1 Plant material

The 40 accessions of weedy rice used in this study were collected in the Piemonte region in Northern Italy and have been described earlier (Grimm et al., 2013). The collected caryopses were planted in the greenhouse of the Botanical Garden of the Karlsruhe Institute of Technology (KIT) in October 2010 at a day temperature of 25°C ± 3°C, 70% ± 15% humidity, and a 16 hr light: 8 hr dark cycle with 2,500 ± 350 µmol m^−2^ s^−1^ photosynthetically available radiation. Seeds were harvested in June 2011; the caryopses of different plants from one accession were pooled. The cultivated Italian varieties were kindly provided by Ente Nazionale Risi (Milan, Italy), and cultivated and harvested in parallel. To get insight into the origin of the *sd1* alleles, additional 12 landraces from India were also included in the study. All seeds were stored at 7°C until use. For details on all accessions used in this study see Table S1 (accessions from Italy) and Table S2 (landraces from India).

### 2.2 DNA extraction

DNA was extracted following the protocol by Doyle (1987) with minor modifications. All chemicals used were obtained from Carl Roth (Karlsruhe, Germany). A small sample (100 mg) of leaf tissue was frozen in liquid nitrogen and ground to a powder (TissueLyser, Qiagen, Hilden, Germany) for 20 s at 21 Hz, before adding 700 µl of CTAB buffer (67°C, 3% w/v cetyl trimetylammonium bromide, 1.4 M NaCl, 0.3 M Tris‐HCl, 25 mM EDTA, pH 8.0), and proteinase K (50 ng/µl). This mixture was incubated at 20°C for 10 min, samples were centrifuged for 5 min at 2,400 *g*, and the upper phase was mixed with 700 µl of chloroform:isoamyl alcohol (24:1). After a further incubation for 10 min at 20°C, followed by centrifugation at 2,400 *g* for another 10 min, the upper phase was transferred into a fresh tube, to which 0.1 volumes of 3 M LiCl and 0.6 volumes of ice‐cold isopropanol were added. The mixture was kept at −20°C for 1 hr, and then spun at 5,400 *g* for 10 min. Precipitates were washed with 70% (v/v) ethanol and dried in a Speedvac concentrator (Eppendorf, Hamburg, Germany). The DNA was dissolved in 100 µl of sterile water, and concentration and purity were determined spectrophotometrically (Nanodrop, Thermo Scientific, Karlsruhe, Germany).

### 2.3 PCR amplification of target genes

Unless stated otherwise, oligonucleotide primer pairs used in this study were designed using the online software Primer 3 (http://primer3.ut.ee/, Untergasser et al., 2012) against the *O. sativa* reference genome published in the Rap database (www.rapdb.dna.affrc.go.jp; Kawahara et al., 2013; Sakai et al., 2013). All primer sequences were checked, before use, against the *O. sativa* reference genome by the BLASTN algorithm to verify that there were no possible alternative binding sites. Primer pairs used for the analysis of the *Rc* gene were selected from Gross, Steffen, and Olsen (2010). Oligonucleotides were synthesized by Sigma‐Aldrich. Sequences, T_m_, fragment length, PCR specification, and references are shown in Table S3.

Fragments were amplified using either standard PCR, or touchdown PCR protocols based on the GoTaq Flexi Kit (Promega). Reactions were carried out in sample volumes of 15 µl with total concentrations of 1× buffer, 75 ng template DNA, 0.8 pmol of each primer (forward and reverse), 2 mM of dNTP mix, and 0.06 units of GoTaq Flexi polymerase, in case of standard PCR. For touchdown PCRs, the same concentrations were used, but 0.5 M betaine and 1.5 mM DMSO were added to the reaction mix. Amplifications were carried out in a Tetrad 2 Engine (Bio‐Rad) with the following program settings: 95°C for 5 min, 35 cycles of denaturation at 95°C for 30 s, annealing at 59°C for 30 s, synthesis at 72°C for 2 min, and a final elongation step of 72°C for 10 min in case of the standard PCR. For touchdown PCR, the program settings were: 95°C for 5 min, 10 cycles of 96°C for 30 s, annealing for 30 s (starting at 59°C and decreasing 0.7°C per cycle), elongation at 72°C for 2 min, followed by 35 cycles of 95°C for 30 s, annealing at 52°C for 30 s, elongation at 72°C for 2 min, and a final elongation step of 10 min at 72°C.

### 2.4 Library preparation and sequencing

For each accession, libraries were constructed and barcoded using the Nextera XT library preparation kit and the Nextera XT indexing kit (Illumina) following the instructions of the manufacturer with minor modifications. For the library preparation, only 25% of the recommended amount of reagents were used, and the libraries were purified using the Agencourt AMPure system (Beckman Coulter). Libraries were quantified by performing the dsDNA HS assay on a Qubit system (Invitrogen, Thermo Fisher Scientific). Library quality was further assessed by running 5 µl on a 1.5% agarose gel (40 min, 100 V). Only libraries for which the majority of fragments was at least 300 bp in length were processed further. Libraries were pooled to equal concentrations (ng/µL) and sequenced on a MiSeq (Illumina) at the Georgia Genomics Facility (University of Georgia).

### 2.5 Data analysis and structural prediction

Raw sequencing reads for each accession were aligned using Bowtie 2.0 (Langmead & Salzberg, 2012) against the *O. sativa *ssp.* japonica* reference sequence (Kawahara et al., 2013; Sakai et al., 2013) accessible at the Rap DB website (www.rapdb.dna.affrc.go.jp). Default settings were used for indexing of the reference and alignment of the reads. The resulting.bam files were sorted using the SAMtools software package (Li et al., 2009) by applying the sort command. Sorted files were visualized using the IGV tool (Thorvaldsdóttir, Robinson, & Mesirov, 2013). Coverage and SNPs were recorded manually for each gene in each accession. The *indica* reference genome was accessed through the public database http://www.mbkbase.org/R498/ indica. The three‐dimensional structure of the *japonica* rice SD1 protein (Gibberellin 20 Oxidase, UniProt accession Q0JH50) was accessed through the ModBase database (https://modbase.compbio.ucsf.edu), domains were annotated by the Pfam database (http://pfam.xfam.org), and the three‐dimensional views were generated using the Swissmodel tool in the ExPASy database (https://swissmodel.expasy.org/repository/).

### 2.6 Phenotyping

Data on plant height used to determine the allelic status at the *sd1* locus were pooled over all accessions of weedy rice and compared to the average height of cultivated varieties. Height data were collected as part of the study reported by Fogliatto et al. (2012). The difference of the respective mean values was tested for significance by a two‐tailed Student's *t* test. The concentration of proanthocyanidin extracted from weedy rice caryopses was estimated by the vanillin assay (Price, Hagerman, & Butler, 1980) with minor modifications. Seeds (1 g) where dehusked and ground on ice using mortar and pestle, mixed with 10 ml absolute methanol and incubated for 1 hr on an orbitary shaker at 100 rpm and 20°C. The debris was separated by centrifugation (10 min, 3,000 *g*), and 1 ml of the supernatant was used for analysis. A standard curve of (+)‐catechin in methanol (0–0.3 mg/ml) was used for calibration. All samples were prepared in duplicate. One set was used for normalization, and 5 ml of 5% concentrated hydrochloric acid was added to these samples. The second set was used for the actual measurement, and 5 ml of 0.5% w/v vanillin in 5% concentrated hydrochloric acid was added to these samples. Samples were incubated for exactly 20 min at exactly 30°C in a water bath (the precision is essential to ensure validity of the results) and, subsequently, the absorption at 500 nm was recorded photometrically (Uvikon XS, Uvikon). The values of the vanillin measurements were normalized by subtracting the values of the corresponding HCl blank controls. A standard curve for catechin concentration was calculated based on the measurements of the (+)‐catechin standards. A linear regression was performed on the data and the equation of the regression line was used to calculate the catechin concentration in the weedy rice samples in mg per gram grain. The results were then converted into µmol catechin per gram grain. To investigate whether variation in catechin concentration was associated with the presence of a specific *Rc* allele, the mean catechin values for the accessions pooled over *Rc* haplotype 1 or 2 were determined and differences were tested for significance by a two‐tailed Student's *t* test. To assess the morphology of the abscission zone, the caryopses were cut transversely close to their lower end and glued onto glass slides such that the abscission zone was pointing upward. The abscission zones for five seeds per accession were recorded with a stereo microscope using a digital image recording system (Leica M420, Leica, Solms, Germany) at 32× magnification. The images were grouped into three categories (rough, intermediate, smooth) depending in the surface texture and relative frequencies of these categories in each accession.

## 3 RESULTS

To facilitate visualization of the cross‐correlations between genetic marker and phenotype, we have organized our results by trait.

### 3.1 Plant height and *semi dwarf1*

**FIGURE 1:**
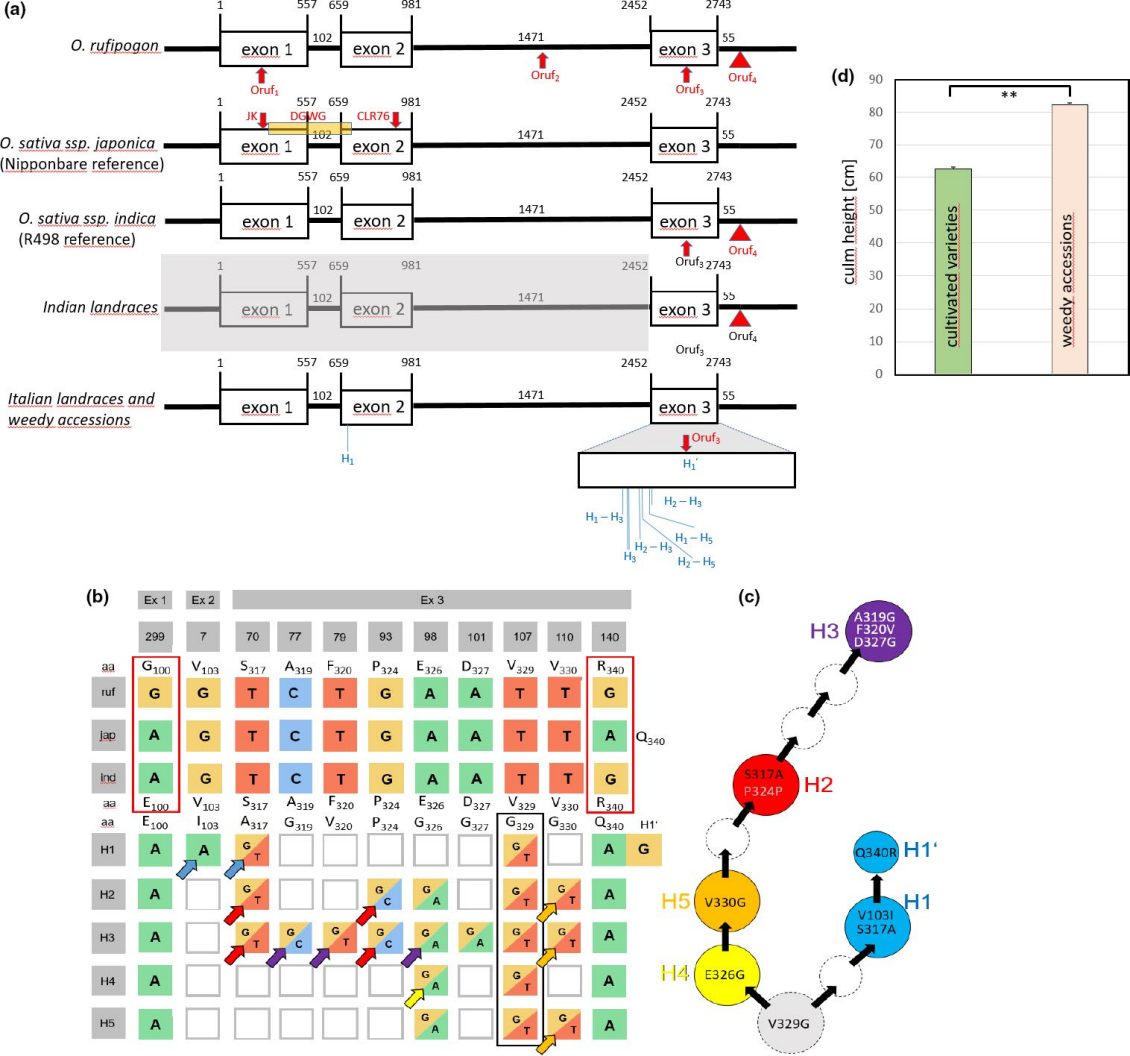
Structural and functional features of the locus *SEMIDWARF 1 (SD1)* for Italian and Indian accessions of rice. (a) Genomic structure of the *SD1* locus in *O. rufipogon* (strain W1843); *O. sativa *ssp.* japonica* cv. “Nipponbare” (reference genome for *japonica*); *O. sativa *ssp.* indica* cv. “R498” (reference genome for *indica*), landraces from India, and Italian cultivated landraces and weedy rice with different alleles found in haplotypes H_1_–H_5_ across Italian cultivated landraces and weedy accessions. H_1_′ is a variant of H_1_ bearing a *rufipogon* signature in exon 3. Numbers give positions in basepairs from the start codon. Signatures specific for *O. rufipogon* (Oruf_1_‐Oruf_4_) are indicated by red arrows, the red triangle Oruf_4_ indicates the position of a characteristic 11‐bp deletion downstream of the stop codon. In the *japonica* allele, positions of the mutations in the *sativa SD1* alleles in Jikkoku (JK), Calrose 76 (CLR76), and Reimei, as well as the deletion in Dee‐Geo‐Woo‐Gen (DGWG, orange box) are indicated (Sasaki et al., 2002). (b) Haplotype map showing the single nucleotide polymorphisms (SNPs) and the resulting amino‐acid residues (aa) in the Italian haplotypes H_1_‐H_5_, compared to *O. rufipogon* (ruf), *O. sativa *ssp.* japonica* (jap), and *O. sativa *ssp.* indica* (ind). Red rectangles highlight differences between the three alleles, note that ind is equal to jap in exon 1, but equal to ruf in exon 3, the black rectangle highlights a SNP present in all Italian haplotypes (both cultivated or weedy). Colored arrows refer to the most parsimonious model (shown in c) explaining these haplotypes. (c). The respective amino‐acid substitutions and the position of the respective residue is indicated. (d) Culm length of cultivated (green bar) and weedy (pink bar) accessions collected in Italy. The difference is significant at *p* = .009. Data represent mean values and standard errors from at least 100 individuals

Since plant height is one of the main traits discriminating weedy rice from cultivated varieties, we sequenced the coding region (three exons) of *SD1* locus in our accession set to identify mutations that might explain the height difference. The obtained sequences were compared to the reference genomes for *O. sativa *ssp.* japonica* (from cv “Nipponbare”) and *O. sativa *ssp.* indica* (from cv “R498”), as well as to the genomic sequence of *O. rufipogon*. To get further insight into the origin of the *SD1* locus alleles present in Italian cultivated varieties, the third exon with the flanking 3′ region was also investigated in 12 common Indian landraces, as such landraces had been the source of seed material for rice cultivation before Italy initiated its own breeding program in the early 18th century. The structure of the *SD1* locus gene, as well as the major differences between the alleles identified in the set of germplasm analyzed are shown in Figure 1a; the corresponding single nucleotide polymorphisms (SNPs) are highlighted in Figure 1b.

Two SNPs included nonsynonymous substitutions which differentiate *O. rufipogon, O. sativa *ssp.* japonica* and *O. sariva *ssp.* indica* (Figure 1b, red rectangles): A transition from a guanine (*O. rufipogon*) into an adenine (*O. sativa *ssp.* japonica* and *O. sativa *ssp.* indica*) in the first exon leads to a substitution from glycine by glutamate at position 100 of the protein sequence. Likewise, in the third exon, an arginine residue present at position 340 in the *O. rufipogon* protein is substituted by a glutamine. Interestingly, the allele does not show this substitution and, thus, is equal to the *rufipogon* allele. Several SNPs are seen in the very long second intron between different accessions of *O. rufipogon*, that otherwise is not altered in length. Downstream of the stop codon, an 11‐bp deletion is found in *O. rufipogon*, but absent in *O. sativa *ssp.* japonica* (Figure 1a, Oruf_4_). Interestingly, this motif, as well as the R_340_ residue, is also found in the *indica* reference genome, as well in all, except two, of the tested 13 landraces from India (Data S1). The only two accessions lacking this motif and, thus, resembling the *sativa *ssp.* japonica* allele, were Basmati and Paw San. This *rufipogon* signature (indicated as haplotype H_1_′) is also found in several Italian cultivated varieties (Figure S1).

In addition to the *japonica* allele, a total of six different haplotypes were identified in the Italian pool of accessions (Figure 1b). Haplotypes H_1_–H_5_ were found in, both, weedy rice and cultivated genotypes, while H_1_′ was only seen in cultivated genotypes, such as Thaibonnet, Creso, Gladio, or Artiglio (Figure S1). Except for one SNP present in haplotype 1 in exon 2, position 7 (leading to a structurally conservative substitution of a valine by an isoleucine residue), all identified SNP were clustered in a small region (40 bp) of exon 3 and predicted to be of structural relevance: For example, negatively charged residues, such as aspartate or glutamate, were replaced by glycine. Moreover, this region spans the C‐terminal part of the iron‐binding dioxygenase domain and part of the substrate binding domain (Figure S2) of the gibberellin oxidase. Interestingly, all SNPs detected in exon three were heterozygous with one allele corresponding to the *O. sativa *ssp.* japonica* reference allele. With the exception of haplotype H_1_′, none of the Italian haplotypes carried any trace of a *rufipogon* signature suggesting that all SNPs occurred in a *japonica* type allele. Haplotype H_1_′, however was closely related to haplotype H_1_, but exhibited residue R_340_ characteristic of the *rufipogon* allele.

To test whether these SNPs might be of functional relevance, we aligned the 250 most closely related GA20 oxidases (Data S2). All SNPs were located at highly conserved sites: If there were amino‐acid substitutions, they mostly involved residues with the same chemical properties. In contrast, the SNPs in the Italian accessions usually led to nonconservative amino‐acid substitutions which were not seen in any of the other GA20 oxidase homologues analyzed. There was one exception: a substitution of a valine at position 329 with a glycine. This glycine residue shifts the border of a β‐sheet and widens the substrate‐binding site (Figure S2), and is present in certain Indian landraces of rice (Kasalath UniProt F7J3C8; Kaluheenati UniProt F7J3D5; Muha UniProt F7J3D3). Mean plant height of the weedy accessions was increased by 25% over that seen in cultivated varieties (Figure 1d).

The haplotypes identified in the Italian accessions harbored different numbers of SNPs relative to Nipponbare—while H_1_ and H_4_ differed only by two SNPs from the *sativa* allele, H_3_ showed eight differences. However, all haplotypes shared the same transversion from thymine to guanidine at position 107 of exon 3 resulting in the replacement of Val_329_ by Gly_329_ as seen in the three Indian landraces mentioned above. It is possible to arrange the observed haplotypes in a contiguous sequence of events, whereby one haplotype derives from the other by the accumulation of additional base exchanges (Figure 1c).

When the prevalence of the different haplotypes was mapped on the microsatellite tree obtained earlier (Grimm et al., 2013), the different haplotypes were distributed over different clades. In the clade comprising most of the Italian cultivated varieties (green clade), *japonica* (Q_340_) and *rufipogon* (R_340_) type alleles were interspersed (Figure S1). The *rufipogon* (R_340_) allele was completely absent from the weedy accessions and found only in a part of the cultigens (Figure 4a).

### 3.2 Seed shattering and *SHA1 (SH4)*

All tested accessions from Italy, no matter whether they were varieties or weedy accessions, harbored a thymine at position 237, which clearly differentiates them from the wild *rufipogon* allele, which shows a guanine. The resulting exchange of a lysine residue in wild rice by asparagine in cultivated rice leads to a loss of function. Because a loss of function means that the seeds will remain on the ear, this mutation is considered a central factor in the transition toward domestication (Li et al., 2006). Our results show that this domestication trait is present in all of the 38 tested accessions from Italy. Although the tested weedy accessions harbored this loss‐of‐function allele, they shed their seeds readily, which required early sampling to avoid loss of seed material (Fogliatto et al., 2012).

**FIGURE 2:**
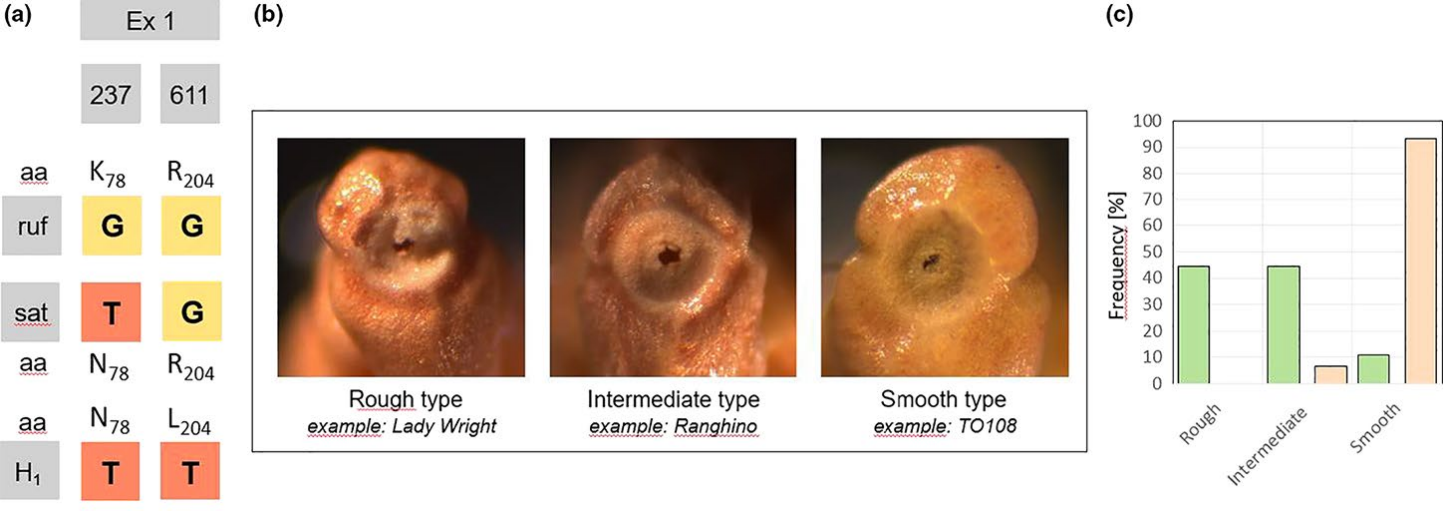
Structural and functional features of the locus *SHA1/SH4* for Italian accessions of rice in comparison to the wild species *O. rufipogon*. (a) Haplotype map showing the single nucleotide polymorphisms (SNPs) and the resulting amino‐acid residues (aa) in the Italian haplotype H_1_, compared to *O. rufipogon* (ruf) and *O. sativa *ssp.* japonica* (sat). The sat haplotype was seen in all 14 tested cultivated accessions, but only in four of the 24 tested weedy accessions. The haplotype H_1_ was found only in the weedy, but in none of the tested cultivated accessions. (b) Classification of the abscission zone into three types depending on the depth of the cavity and the appearance of the surface. In the rough type, the caryopses remain on the ear, while in the soft type, they are readily scattered. (c) Frequency distribution of the abscission‐zone types in cultivated (green) versus weedy (red) accessions from Italy. The histogram is based on a sampled of 18 weedy and 15 cultivated accessions; the classification of each accession is based on microscopic analysis of five individual caryopses per accession

The Italian accessions displayed two different haplotypes (Figure 2a); all 14 tested cultivated accessions, but only four out of the 24 weedy accessions harbored the allele seen in the *O. sativa* ssp. *japonica* reference genome (Figure S1). The vast majority (20 out of 24) of weedy accessions showed a second haplotype (Figure 4b) with a transversion at position 611 of exon one, such that the guanine found in the *japonica* allele was exchanged by a thymine (Figure S1). This would replace an arginine residue at position 204 by a leucine residue. This mutation is located in a region between the tri‐helix DNA‐binding domain and a proline‐rich region and is highly conserved, as seen by the fact that among 100 cultivated and wild rice *SH4* sequences recovered by a BLAST search, only one (SwissProt I3PJ85) was found that shows the same mutation (Data S3). This one sequence was obtained from a weedy accession and the SNP at position 611 has been described by Zhu et al. (2012) as a fixed SNP for weedy rice accessions from Italy and Spain. The replacement of an arginine with a leucine residue is expected to be relevant, because it will cause a change of charge.

We, therefore, investigated the texture of the abscission zone of detached caryopses by stereo microscopy. The morphology of the abscission zones could be classified into three types that were defined as rough, intermediate and smooth (Figure 2b). In rice, similar to other cereals, a rough surface has been shown to be a hallmark of disrupted shattering (Jin, 1986). The impaired formation of the abscission zone causes the seed to remain on the rachis, such that it has to be detached by mechanic force in a nonspontaneous manner (Jin, 1986). In contrast, a smooth surface caused by a fully developed abscission layer is characteristic for accessions with efficient shattering, where caryopses detach spontaneously. The intermediate type represents a transitional situation with a partially developed abscission layer and was prevailing in moderately shattering accessions. Here, shattering was observed, but remained incomplete as compared to smooth phenotypes.

A frequency distribution over these three categories (Figure 2c) revealed that the majority of cultivated genotypes showed either a rough or intermediate type, while a smooth abscission zone was found only in two cases. These exceptions were two ancient landraces, Bertone and Ostiglia, which are no longer cultivated due to their strong tendency for shattering. In contrast to cultivars, more than 90% of the weedy rice accessions displayed the smooth phenotype, while the rough phenotype was completely absent from the weedy varieties.

### 3.3 Pericarp pigmentation

For this study, the region around the characteristic 14‐bp deletion was sequenced and compared to the phenotype, determined by quantification of the proanthocyanidin content in the caryopses.

**FIGURE 3:**
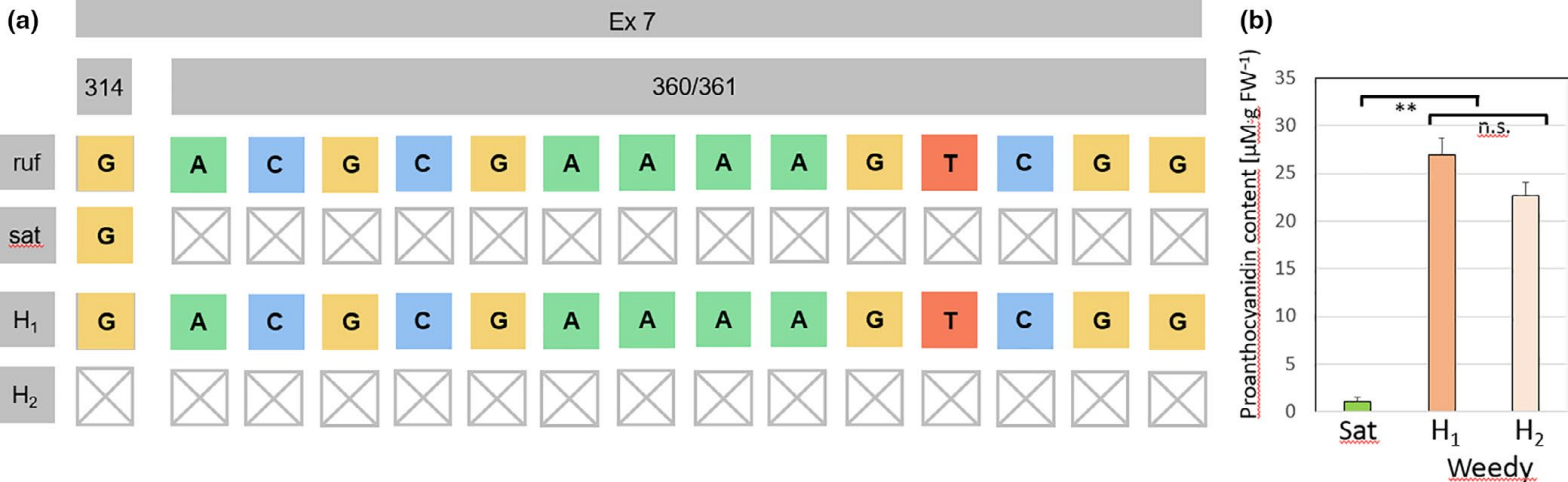
Structural and functional features of the locus *Rc* for Italian accessions of rice in comparison to the wild species *O. rufipogon*. (a) Haplotype map showing the 14‐bp deletion in exon 7 for *O. sativa *ssp.* japonica* (sat) as compared to the allele from *O. rufipogon* (ruf). The sat allele was exclusively seen in the cultivated accessions. In the weedy accessions, two haplotypes were found. Haplotype H_1_ was identical to the *rufipogon* allele and was found in 25% of the tested weedy accessions, but in none of the tested cultivated accessions. Haplotype H_2_ showed the 14‐bp deletion characteristic of the sat allele, but in addition carried a 1‐bp deletion 46 bp upstream. Both deletions combined will restore the reading frame downstream of the 14‐bp deletion and thus likely deliver a largely functional product. This allele was seen in 69.6% of the tested weedy accessions, but in none of the cultivated Italian accessions. (b) Quantification of proanthocyanidin content in the two weedy haplotypes compared to the Italian *O. sativa *ssp.* japonica* cultivar “Arborio” (sat). Haplotype 2 shows a slight decrease, which is, however, not significant. Values represent mean and standard error for 1 g of seed material per accession. Haplotype 1 was represented by 16, haplotype 2 by 4 accessions

**FIGURE 4:**
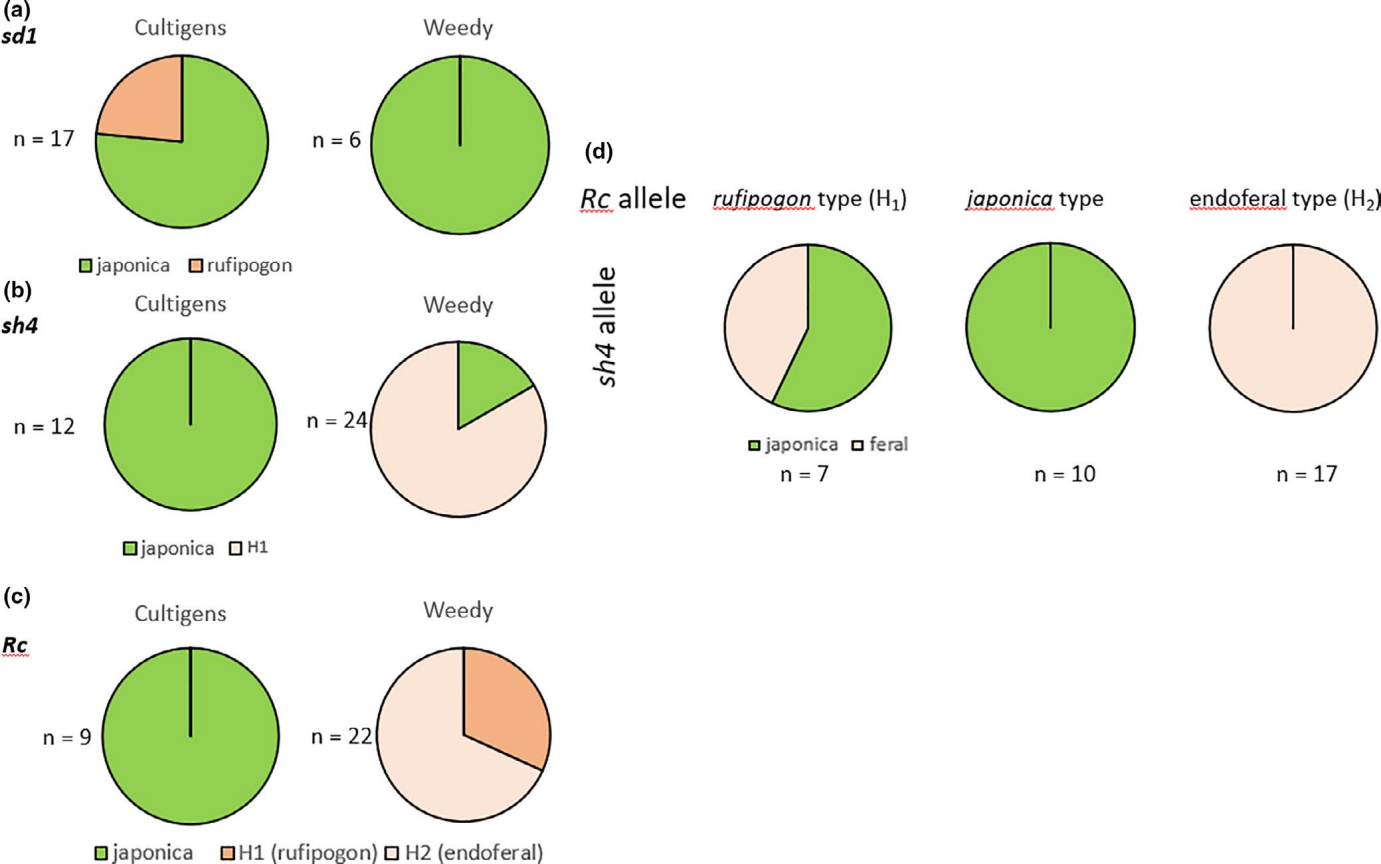
Incidence of weedy alleles in domestication genes of cultivated and weedy accessions of Italian rice. (a) The (more active) rufipogon allele of sd1 is exclusively found in the cultivated accession, while all weedy accessions harbor the *japonica* allele or derivatives of it. (b) All tested cultigens harbor the nonfunctional allele *sh4* from *O. sativa japonica,* the majority of weedy accessions the allele H1. (c) All tested cultigens harbor the japonica allele of Rc linked with the lack of pericarp coloration, while all weedy accessions either harbor the original (functional) *rufipogon* allele or the endoferal allele H2, where reading frame is restored by a second‐site deletion. (d) Coupling of weedy alleles for *Rc* and *sh4* in cultivated and weedy accessions of Italian rice The frequency of the *japonica* and the feral (weedy) allele H_1_ (see Figure 2) in dependence on the three alleles identified for the *Rc* locus. The *japonica* type *Rc* locus is tightly associated with a *japonica* type *sh4* allele, while the endoferal *Rc* allele (H_2_) is tightly associated with a feral *sh4* allele. For a *rufipogon Rc* allele, both *japonica* and feral alleles of *sh4* can occur

We identified three different haplotypes in our sample pool (Figure 3a). Not surprisingly, the *japonica* allele with the characteristic 14‐bp deletion was found in all cultivated varieties, but was absent from all tested weedy accessions (Figure 4c). The weedy accessions, in turn, clustered into two haplotypes. Haplotype 1 lacks the 14‐bp deletion and is identical to the *Rc* allele of *Oryza rufipogon*, the presumed wild ancestor of *O. sativa*. Haplotype 2 shares the 14‐bp deletion with the *japonica* allele, but harbors an additional 1‐bp deletion 46 bp upstream of the 14‐bp gap. The *rufipogon* allele was found in 30.4% of the tested weedy accessions, while the haplotype 2 (derived from the japonica allele) clearly dominated with 69.6% (Figure S1). Due to the second deletion, the reading frame downstream of the 14‐bp gap is reinstalled leading to a protein product that is only 5 amino acids shorter than the *rufipogon* Rc protein. The observation that all accessions with this haplotype showed red pigmentation in the caryopses pericarp, while all accessions with the *japonica* allele were white, is consistent with the regained functionality of this allele. To validate this assumption, we quantified the abundance of proanthocyanidins (Figure 3b). As expected, proanthocyanidins could not be detected in caryopses of cultivars(bearing the japonica allele with the 14‐bp deletion), but the mean value pooled over the accessions from haplotype 2 was reaching almost the same level as that seen in the accessions harboring the full‐length *rufipogon Rc* allele (haplotype 1). A slight reduction of around 15% was not significant (Figure 3b) in a two‐sample *t* test indicative of almost complete functionality of haplotype 2.

### 3.4 Alleles for restored seed coloration and seed shedding are coupled

When the different alleles identified for the three loci (*sd1, sha1/sh4, Rc*) were mapped on a phylogenetic tree generated on neutral SSR markers (Grimm et al., 2013), clear patterns of association between *Rc* and *sh4* emerged (Figure S1):

All of the tested cultivated varieties of Italian rice harbored *japonica* type alleles for *sha1/ sh4* and for *Rc*. This means that none of the cultivated varieties showed any wild or feral footprints for seed shattering or for seed coloration. We did detect, however, wild or feral alleles for the *sd1* locus: four of the 19 tested cultivated varieties (Artiglio, Creso, Gladio, Thaibonnet) showed a *rufipogon* signature for the *sd1* locus, and seven showed one of the endoferal alleles derived from a *japonica* template.

All of the wild or feral alleles for *sha1/ sh4* and *Rc* were exclusively found in weedy accessions (Figure S1, Figure 4d). A *rufipogon* type allele of *Rc* (H_1_) could be seen both with a *japonica* type or with a feral form of *sh4*. An endoferal (red, H_2_) allele of *Rc*, however, was never seen together with a *japonica* type (nonshattering) allele of *sh4*. In other words: if a seed was red due to the restored Rc allele H_2_, it was inevitably shattered.

## 4 DISCUSSION

The current work focused on the genetic and phenotypic aspects of traits that had been central to the domestication of rice in the evolution of weedy rice. The basic motivation was to determine whether the observed de‐domestication of weedy rice was using ancestral “wild” alleles that had been cryptically preserved in the gene pool of domesticated rice (exoferality), or whether ferality was achieved de novo by changes of domesticated alleles (endoferality). Rice cultivation in Italy provides a kind of historical laboratory to address this question, because this agricultural ecosystem developed in the absence of any sympatric Crop Wild Relatives in a historically defined and well‐documented process. In our previous work (Grimm et al., 2013) we had constructed phylogenetic relationship between a large number of weedy accessions, but also old, meanwhile outdated, landraces of Italian rice, and some modern cultivars based on neutral, but highly resolving markers (microsatellites). This revealed that, while weedy and cultigen accessions were mostly clustering to separate clades, some of the historic landraces were found to relate to the weedy rather than to the cultigen accessions. This stimulated the null hypothesis for the current work that weedy alleles of domestication genes might have supported the introgression of weedy traits into these historic landraces. In the following, we will, therefore, first discuss plant height, seed shattering and pericarp pigmentation individually, and subsequently develop an integrated timeline model for the evolution of Weedy Rice in Italy.

### 4.1 A green revolution gene as Trojan Horse for endoferality

One of the easiest ways to spot weedy rice in a paddy is to search for individuals that stand out over the canopy of the cultivated varieties. However, also in traditional cultivated varieties, plants were taller. As in other cereals, reduced plant height has been a central target for breeders, because shorter culms correlate with a higher resistance against lodging. A reduction of culm length by a factor of two will reduce the lever momentum four‐fold (Oda, Suzuki, & Odagawa, 1966). Genes for the biosynthesis of gibberellins as crucial regulators of stem elongation are of prime interest in this context. However, since gibberellins are also needed for flower development and grain filling, global blocking of gibberellin synthesis would not be a feasible strategy. A solution for this dilemma is offered by the key enzyme gibberellin‐20‐oxidase, which converts the inactive precursor GA53 into GA20, from which the even more potent GA1 can be produced (Ashikari et al., 2002). This enzyme is encoded by two loci that are expressed in different tissues: While GA20ox‐1 is active in floral meristems and drives grain filling through modulation of cytokinins (Wu et al., 2016), its isogene, GA20ox‐2, is exclusively expressed in vegetative tissues and is encoded by the *semi dwarf 1* (*SD1*) locus. Several independent mutations in the *SD1* locus resulting in a semi dwarf phenotype kicked off the green revolution in the 1960ies (Monna et al., 2002; Sasaki et al., 2002; Spielmeyer et al., 2002). In our current sample set, none of these mutants was found. It is often overlooked, though, that long before the Green Revolution, namely during the domestication of *O. rufipogon*/ *O. nivara* more than 6,000 years ago, two crucial amino‐acid exchanges (E100G in exon 1 and Q340R in exon 3) had occurred in the ancestral line of *O. sativa *ssp.* japonica* that reduced the activity of GA20 oxidase by about 75% (Asano et al., 2011). Since the Italian accessions of weedy rice were significantly longer than cultivated varieties (Figure 1d), we scrutinized the *SD1* locus as a potential candidate gene related to this phenotypic trait.

Among the loci sequenced in this study, *SD1* was the gene with the highest variability (6 haplotypes) consistent with a functional relevance for a “weedy lifestyle.” This is in line with findings on weedy rice in the US that also report considerable variation at the *SD1* locus (Reagon et al., 2010). We investigated, whether these haplotypes were derived from the ancestral *rufipogon* allele (which would be a hallmark of exoferality), or, alternatively, originated from the domesticated *japonica* or *indica* allele (indicative of endoferality). In fact, we were able to detect characteristic *rufipogon* footprints (a G at position 140 of exon 3 leading to an arginine residue at position 340, as well as a 11‐bp insert 55 bp downstream of the stop codon). This *rufipogon* signature (which is also present in *indica* rice), qualifying as an exoferality mark, was quite rare; however, it was not detected in any of the weedy accessions, and was present in only four of the 19 tested cultivated varieties. These four traditional varieties (Artiglio, Creso, Gladio, Thaibonnet) are long grained and of the *indica* type (https://www.risoitaliano.eu/), while the majority of current Italian varieties are of the *japonica* type. Since seed material was imported from India, before own breeding programs started around 1,800, we sequenced exon 3 of the *SD1* locus from available Indian landraces of rice (Data S1). Our results show that this *rufipogon* signature was predominant in *indica* rice, since among 13 tested varieties only two (Basmati and Paw San Yin) were devoid of this exoferality trait. However, this *rufipogon* signature is not found in *japonica* varieties. Since this *rufipogon/ indica* mark was not present in any of the weedy accessions tested, exoferality of the *SD1* locus seems to be negligible as a driving force for weediness in Italian rice.

A closer look revealed that the haplotypes seen in Italian rice clustered to a very narrow region in exon 3 (Figure 1a). Moreover, they all shared a putative founder mutation leading to a replacement of a valine at position 329 into a glycine (Figure 1b). This glycine residue should shift the border of a β‐sheet and improve the access of the substrate into the binding pocket (Figure S2). While this mutation was not found in any of the *japonica* varieties, and also could not be recovered from any homologues of the *SD1* locus from *O. rufipogon*, *O. nivara* or other wild rice species published in GenBank, it is present in some traditional landraces from India (Kasalath SwissProt F7J3C8; Kaluheenati SwissProt F7J3D5; Muha SwissProt F7J3D3). It, thus, qualifies as an endoferality mark, which might reinstall a higher activity of GA20 oxidase, and therefore compensate for the loss of activity that had occurred during rice domestication. It would be interesting to test, whether the recombinantly expressed gene product encoded by the H_1_ allele shows a higher enzymatic activity in vitro compared to its *japonica* counterpart in a similar way as was done for the *rufipogon* allele (Asano et al., 2011). In fact, for a genotype belonging to the landrace Kasalath, gibberellin levels in etiolated coleoptiles have been found to be elevated by around three‐fold as compared to the *japonica* variety Nihonmasari (Toyomasu, Yamane, Murofushi, & Nick, 1994), indicative of a higher activity of the H_1_ allele. It is possible to integrate all haplotypes into a parsimonious scenario, where, starting from an ancient Indian template with a Gly_329_, the haplotypes found in our Italian sample set can be derived by a sequence of additional mutations (Figure 1c). In this scenario, haplotypes H_1_ and H_4_ would represent a primordial state, while H_3_ would derive from the accumulation of additional mutations. Interestingly, haplotype H_1_ centered around the cultivated varieties of rice (Figure S1). Since it is also seen in Italian varieties which display a typical *japonica* phenology, such as broad grains rich in amylopectin (for instance, the economically important variety Arborio, registered in 1946, which is classically used for *risotto*), it might be interpreted as a genetic footprint from the time before the initiation of Italian breeding programs, when seeds were regularly imported from India.

The scenario emerging from this analysis shows two levels of ferality: while the functionally more active (Asano et al., 2011) ancient *rufipogon* allele of the *SD1* locus is dominant in Indian landraces (exoferality), it did not play a role in the genesis of weedy rice in Italy, but remained confined to long‐grained varieties that are of the *indica* type. In contrast, a founder mutation (Val329Gly) that probably occurred in a lineage of Indian landraces, and possibly reinstalled GA20 oxidase activity (Toyomasu et al., 1994) has been the driving force for a whole group of variants that are spread over several lineages of Weedy Rice (Figure S1). This founder mutation and its derivatives would therefore qualify as endoferality trait (whereby the process of endoferality had already begun in India and continued after seed transfer to Italy). The fact that haplotype H_1_, which seems to be the closest recent derivative of the putative ancestor seen in the Indian landrace lineage, is focused on the cultivated accessions, would further support an endoferal scenario, where this allele was passed on from cultivated ancestors to their weedy progenies. Since these endoferal alleles of the *SD1* locus are likely to confer elevated (“weedy‐type”) elongation, this resulting phenotype would be subject to two antagonistic selection pressures: the faster growth allows to outcompete cultivated varieties, but on the other hand also facilitates detection by humans. Antagonistic selection will in the long run provide a fitness benefit for the heterozygotes that can escape human selection, but will give rise to more competitive offspring, which might be the reason why the majority of weedy accessions were heterozygous for the *SD1* locus. This is not the first time that heterozygosity of the *SD1* locus had been reported for weedy rice: this phenomenon has also been reported for weedy rice in the US (Reagon et al., 2010), but at a low frequency. The difference might be related to the fact Reagon and colleagues used accessions that had been cultivated for over 30 years in the Dale Bumpers Rice Research Institute through inbreeding, which should reduce heterozygosity, while in our study samples were collected directly from the infested paddy.

As a summary of our conclusions on the *SD1* locus, we can state that exoferality (i.e., introgression by alleles originating from wild ancestors of rice), although detectable, did not play a role in the evolution of Italian weedy rice. Instead, endoferal processes (i.e., mutations in domestication‐related genes leading to a restoration of the ancestral wild trait) seem to be more relevant. These endoferal processes have possibly already initiated in the Indian germplasm contributing to the breeding of Italian varieties. These endoferal traits were probably passed on in the heterozygous state, because human selection acted against the expression of weedy traits (such as seed shed, coloration of the pericarp, or hypertrophic elongation of seedlings). The re‐introduction of direct seeding (Ferrero & Vidotto, 2010) and the spread of semi dwarf varieties undermined the efficiency of this human selection, such that the *SD1* locus, one of the major drivers for Green Revolution, diversified into new alleles that promoted a weedy behavior, but remained unnoticed, because they were not phenotypically manifest due to heterozygosity. Outcrossing rates in rice are dependent on environmental conditions, but are in the range of up to 10% (Phan, Kageyama, Ishikawa, & Ishii, 2012), which is not very high, but would be sufficient to introgress a weedy allele into a cultigen population. To use a metaphor from Greek mythology: The *SD1* locus became the Trojan Horse, on (or better in) which weedy rice could conquer Italian rice paddies. This development is still fairly recent. Still end of the 1950ies, every year around a quarter of million workers (so called *mondine*) were transplanting (and at the same time weeding) rice in the Italian paddies (ENTE Risi, 2012). But already in the 60ies, direct seeding had replaced this practice already completely (Francese, 2017).

### 4.2 Seed shattering, a central domestication trait as target for endoferality

Seed dispersal is key to the survival of wild species and therefore under tight genetic control. At the developmental level, seed shattering is linked to the development of abscission zones, a process that is orchestrated by transcription factors. For rice, the locus *SHA1/SH4* encoding a tri‐helix transcription factor has been recognized as central (Li et al., 2006). A point mutation leading to an exchange of a positively charged lysine at position 78 by a noncharged asparagine has been identified as a hallmark of domestication. This highly conserved lysine is thought to be essential for DNA binding, and the mutation, which is found in all tested domesticated varieties of *japonica* and *indica* rice (Lin et al., 2007), results in loss of function of this transcription factor, such that the abscission layer cannot be formed and the caryopses remain on the ear. This mutation, which would be eliminated by natural selection, has been positively selected for during the domestication of rice. Different alleles of the *SD1* locus can persist, albeit with different levels of success under conditions of a wild (or likewise, a weedy) lifestyle, as well as under human selection in agricultural ecosystems. In contrast, the activity or loss of function of *SHA1/SH4* represents more or less an all‐or‐none decision—when the seeds are shed, they are lost for human use; when they are not shed, the plant will not be able to propagate without human assistance by sowing. However, other studies challenge this mono‐causal model, since shattering in weedy rice has been observed despite the fact that the respective accessions have fixed the nonfunctional allele (weedy rice in the US: Thurber et al., 2010; weedy rice in China: Zhu et al., 2012; Qiu et al., 2014; our study). On the other hand, Yan et al. (2017) showed that even partial silencing of *SH4* expression resulted in reduced shattering. Our finding that some of the weedy rice accessions shatter their seeds, despite the presence of the “nonshattering” allele (Figure 2a), therefore merges into results from weedy rice populations collected in other regions of the world. It should be noted, however, that only a minority (around 15% in our study) of the weedy accessions fell into this category (Figure S1).

The overwhelming majority of weedy accessions displayed haplotype H_1_ which is derived from the nonshattering allele by one additional point mutation, G611T, in exon 1, which will cause the highly conserved charged arginine residue 204 to be exchanged for a uncharged leucine (Figure 2a). This allele has previously been identified as a fixed SNP in Italian and Spanish accessions of weedy rice (Zhu et al., (2012), but seems to be absent from *O. rufipogon*. Whether this amino‐acid substitution in the linker region between the tri‐helix DNA‐binding domain and a proline‐rich region might be able to restore the impaired DNA binding of the nonshattering gene product remains to be elucidated. Due to the lack of three‐dimensional templates for this family of transcription factors, one would need to do gel‐shift assays with recombinant protein to get insight into this issue.

We therefore used a different strategy and investigated the surface structure of the abscission zone as phenotypic readout for activity. Accessions with pronounced shattering were characterized by a smooth abscission zone, while a rough abscission zone was found in nonshattering phenotypes (Figure 2b). Between these extremes, many accessions displayed intermediately developed abscission zones, correlated with a moderate degree of scattering (Figure 2b). Domesticated accessions had a rough or intermediate abscission zone, as expected for an absence or a mild degree of shattering (Figure 2c). However, two cultivated rice varieties with a smooth abscission zone (indicative of easy shattering) were identified. These were the old landraces Bertone and Ostiglia that, based on our SSR‐based phylogeny (Grimm et al., 2013), were considered likely ancestors of weedy rice. Interestingly, both landraces, as well as a substantial proportion of the other cultivated varieties that showed an intermediate abscission zone, indicative of moderately shattering, harbored the nonshattering allele. Moderately shattering genotypes might have been preferred over nonshattering varieties in the past because hand‐threshing could be performed much easier on those varieties (Ji et al., 2006; Li et al., 2006). This fits with the fact that our sample set contained many traditional varieties that were in use during the 19th century but are meanwhile outdated. These genotypes displayed moderate shattering during propagation in the greenhouse. These patterns of incidence are not compatible with the concept of a mono‐causal relationship between loss of function of *SHA1/SH4* and suppression of shattering, but calls for a modulating role by other genetic factors such as *SHAT1* (Zhou et al., 2012), or *qSH1* (Onishi, Takagi, Kontani, Tanaka, & Sano, 2007). In contrast, the smooth abscission zone seen in most accessions of weedy rice correlated with the presence of haplotype H_1_, that is, the possibly reconstituted allele of *SHA1/SH4*.

Irrespective of the question to what extent the reconstituted shattering in the weedy accessions can be attributed to the H_1_ allele of *SHA1/SH4*, the fact that all the weedy accessions studied here carry the domestication footprint G at position 237 in exon 1 shows clearly that Italian weedy rice derives from endoferality. Again, a central domestication trait has been hijacked by a mutation that is fixed due to selective pressures that are antagonistic with human selection.

### 4.3 The third Trojan Horse: The *Rc* locus as target for exo‐ and endoferality

While seed dispersal represents a switch deciding between a “wild” (or “weedy”) versus a “domesticated” lifestyle, seed coloration, at first sight, does not display obvious links with domestication. In fact, there exist numerous landraces of Indian rice, where the pericarp is pigmented by proanthocyanidins. Nevertheless, the transition from red to white pericarp was a distinct historic event in the domestication of *japonica* rice and is linked to loss of function of the *Rc* locus on chromosome 7. This transcription factor regulates the proanthocyanidin synthesis pathway and underwent a 14‐bp deletion in exon 7 leading to a shift of the reading frame (Sweeney et al., 2007). The frameshift culminated in a premature stop codon and loss of function. As a result, all *japonica* varieties are white. The observation that seed shed and pericarp pigmentation are highly associated in the weedy accessions (Figure 4) indicates an important role of proanthocyanidin in the “wild/weedy” lifestyle. In other words: if a seed is shed, it has to be red. A functional link might be the pronounced antimicrobial activity of proanthocyanidins (reviewed in Winkel‐Shirley, 1996) as a necessary precondition for the pronounced dormancy of weedy rice seed. On the other hand, it is this pericarp pigmentation that is used by humans as the main tool to select against weedy rice. Consequently, red‐seeded variants are selected against since seed producers are obliged to check samples of their seed stocks for the absence of pigmented caryopses. In our study, we could identify three alleles by sequencing the regions neighboring the 14‐bp deletion present in *japonica* rice (Figure 3a). As expected for the very stringent selection against colored caryopses during seed production, the typical cultigen allele for white pericarp with the 14‐bp deletion was found in all cultivated varieties. The complete absence of this allele in the weedy rice accessions is consistent with a crucial function of seed coloration for a “weedy lifestyle,” where shed seeds can remain dormant in the mud, resisting to microbial attack over a long time (Figure S1). Such a correlation between the state of the *Rc* locus and dormancy has also been observed in a global study on pericarp pigmentation in rice (Sweeney et al., 2007). Recent studies showed that the *Rc* plays an important role in the control of seed dormancy and longevity in weedy rice (Pipatpongpinyo et al., 2020).

The discovery of two alleles linked with red coloration points to a scenario in which exo‐ and endoferality act in concert: the functional *rufipogon* allele (haplotype H_1_, Figure 3a), which was found in around 30% of the weedy accessions, is a clear indicator for introgression from wild rice. Since there exist no Crop Wild Relatives for rice in Europe, the most straightforward scenario is that this allele was “imported” during the several centuries before 1,800, when seeds were imported from India. These seeds were either contaminated with wild rice or, alternatively, seeds of one of the common pigmented Indian landraces, were imported (such that the introgression event would have occurred already in India, similar to the situation for haplotype H_1_ for the *SD1* locus). In contrast, the second haplotype (H_2_), which derives from the nonfunctional precursor, is a clear suppression mutation, where the reading frame was reinstalled by a second 1‐bp deletion 43‐bp upstream of the 14‐bp gap. The resulting protein, predicted to lack five amino acids, seems to be functional as seen from proanthocyanidin levels comparable to those seen in haplotype 1 (Figure 3b). This haplotype, dominating in the Italian weedy accessions, is therefore a clear endoferality mark. The fact that this mark cannot be found either in our set of Indian landraces, nor in sequences from wild rice available in public databases, indicates that this endoferality event occurred in Italy itself. The same allele has been described in the context of the transition from the white Italian variety Perla into the spontaneously arising colored derivative Perla‐Rossa (Gulick, Lee, Lupotto, & Powell, 2009). After screening additional varieties, we could also detect it in the outdated landraces named after their origin as Borgo Vercelli, Terranova, and Villanova. It is interesting to note that a similar case had been reported for the US, where the shift of the rice cultivar Wells into the colored Red‐Wells was found to correlate with a single deletion of 1 bp which also restores the reading frame of the nonfunctional Rc allele (Brooks, Yan, Jackson, & Deren, 2008). However, this American allele comes from a different event, because this second deletion is located not 43 bp, but 19 bp upstream, of the characteristic 14‐bp gap.

For the *Rc* gene, we therefore arrive at a scenario whereby the *rufipogon* allele was introgressed in a historic exoferality event, while the suppression mutation has been contributed by ongoing endoferality.

### 4.4 A timeline model for the evolution of weedy rice in the Italian Piemonte region

**FIGURE 5:**
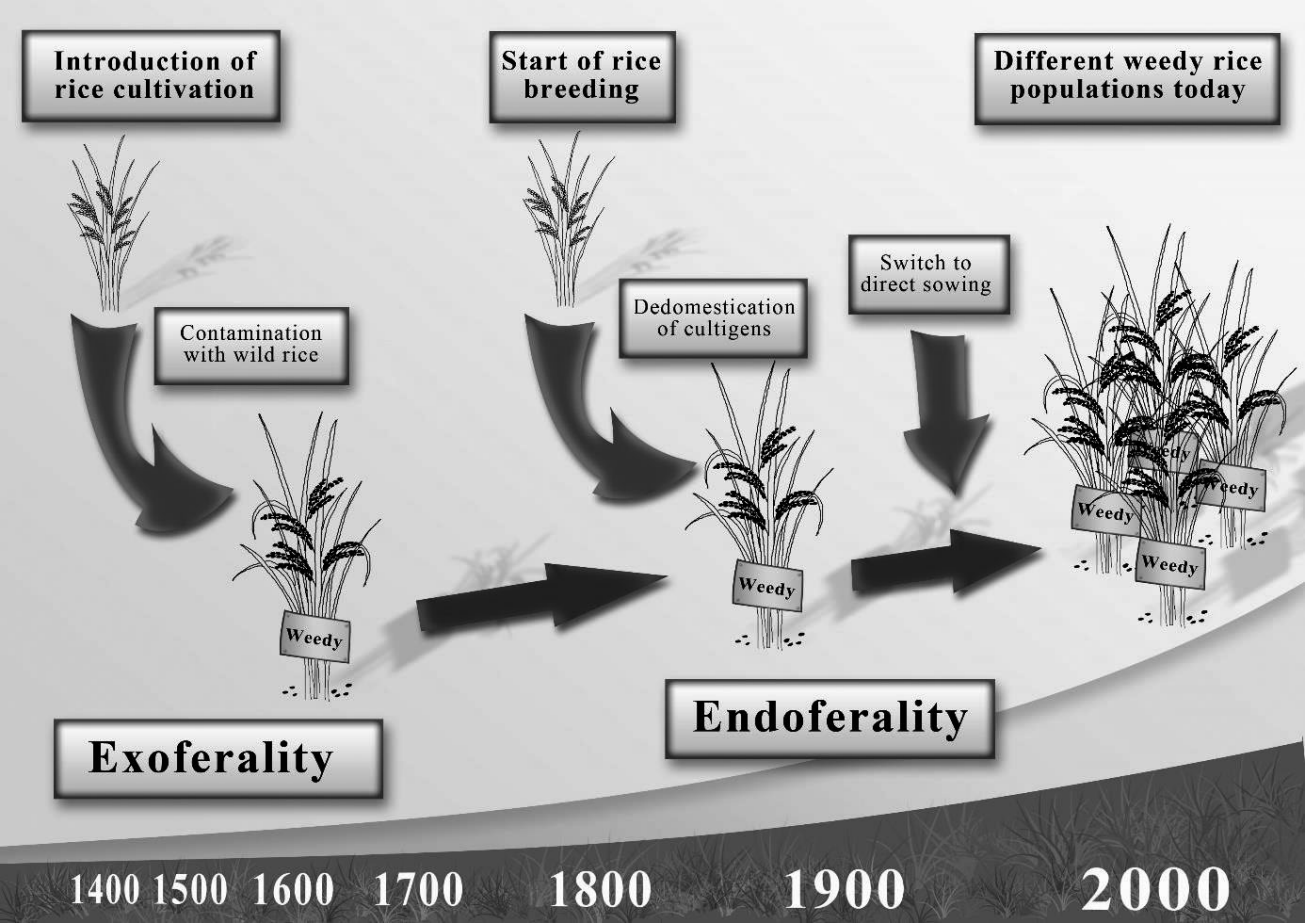
Timeline model for the evolution of weedy rice in Northern Italy. The data generated by this study suggest that contaminated seed stocks imported from Asia introduced *rufipogon* alleles for domestication traits (most prominently, for pericarp pigmentation). This step represents an exoferality event. With the establishment of rice breeding programs in Italy around 1,800 until today, several de‐domestication events of cultigens produced novel weedy traits by endoferality. These spread to a certain extent, partially in the heterozygous state (e.g., the *sd1* locus). With the switch from transplanting to direct sowing in the 1960s, the selective pressure for domestication alleles was drastically reduced leading to an increase in infestation rates

The results of our previous investigation (Grimm et al., 2013) and the current study reveal multiple events that shaped and continue to shape the population structure of weedy rice in northern Italy as it is found today. Therefore, our data complement a study from Huang et al. (2017) showing that weedy rice is an example of recurrent evolution in South Asia and the US, by adding a European case. Our data strongly support both, endoferality (de‐domestication) and exoferality (introgression of wild rice), as mechanisms in the evolution of Italian weedy rice. By linking those data to historic data of rice agriculture in Italy, we arrive at a working model for the development of weedy rice in the Piemonte region of Italy from the beginnings of rice farming in the area in the 15th century until today (Figure 5). The main purpose of this model is to structure future research, such as the search for private SNPs, or the estimation of divergence times between weedy and cultigen accessions.

Rice farming in Italy is estimated to have initiated in the 15th century (Faivre‐Rampant et al., 2011). Originally, seed material was imported from Asia and cultivated in paddies. The exoferality events through wild rice, most likely *O. rufipogon* as suggested by the investigation of the *Rc* gene, most likely date back to that time. Wild rice from Asia or landraces carrying genes from its feral ancestors could have reached Europe as contamination in seed stocks, and wild genes could have entered the gene pool on that route and started the first wave of weedy rice spreading. Also, during that time, rice was broadcast seeded, limiting manual weed control and paving the way for the first wave of weedy rice populations (Ferrero & Vidotto, 2010). When, from the early 19th century, the Italian breeding program became successful, the import of seed stocks from Asia turned obsolete and vanished over time. Those first landraces had a considerably higher genetic variability than the genetically homogeneous modern cultivars. This level of genetic diversity in the cultivated varieties allowed for evolutionary change. In fact, the three traditional landraces popular in the 19th century (Bertone, Ostiglia, and Ranghino) included in our studies had been shown to be closely related to some populations of Weedy Rice (Grimm et al., 2013). We hypothesize that a second wave in the evolution of weedy rice was driven by de‐domestication of these or related landraces. Interestingly, the contemporary varieties Flipper are also genetically closely related to weedy rice, suggesting that the formation and evolution of weedy rice populations in Italy is a continuous and ongoing process. Starting from the 1910s, the practice of transplanting rice was introduced in Italy which greatly reduced the weeding labor and led to better control of weeds and became the most adopted practice. When, in the 1950s, the use of herbicides was adopted in Italian rice farming regions, transplanting was almost entirely replaced by direct seeding on flooded soil (Ferrero & Vidotto, 2010). This switch in the sowing practice removed one of the most important control strategies for weedy rice, promoting the outbreak of infestations and new populations of weedy rice. The link between direct seeding as dominant rice establishment method and severe infestations with weedy rice has been reported for several countries (Singh et al., 2013).

A similar scenario for weedy rice evolution was recently reported by De Leon et al. (2019), who found evidence that one source of weedy rice in California has most likely developed outside of California and was imported, and Hoyos, Plaza, Li, and Caicedo (2020) who showed in a study that Colombian weedy rice has evolved from two scenarios, being accidentally imported from the US and by de‐domestication of local cultivars.

### 4.5 Domestication and de‐domestication—An evolutionary perspective on weed management

As to be concluded from our data, weedy rice invaded and spread in the study region over 500 years, and formed several genetically distinct populations, a process which is fast compared to other timescales in evolution. The multiple origins (exoferal and endoferal) of weedy rice in Italy probably occurred at different time points. For instance, the presence of the H_1_ allele of the *sd1* locus in certain Indian landraces indicates that this is an Indian endoferality event and the respective allele had been “imported” to Italy, while the absence of the H_2_ allele of *Rc* from any of the tested Indian landraces suggests a genuinely Italian endoferality event.

Weed evolution is often described by a co‐evolutionary arms race between humans and weeds. The more stringently humans select against weedy traits, the faster the weed will alter those traits to circumvent selection. As result of these antagonistic selective forces, both sides (weeds and human agriculture) will run faster, just to remain where they are, a situation termed, in allusion to Alice in Wonderland. “Red‐Queen Dynamics” (Neve, Vila‐Aiub, & Roux, 2009; Van Valen, 1973). Whether weed management will be sustainable will depend on the extent to which the respective strategy can escape this co‐evolutionary context. The two forms of ferality differ, however, with respect to the origin of de‐domestication alleles. In exoferality, a domesticated crop turns into a weed by integrating alleles from crop wild relatives. In endoferality, a crop develops weedy traits from de novo mutations in domestication loci. Endoferality differs from exoferality in that the “Red Queen” deals with maintenance of a weed, rather than with the reasons that turn a domesticated crop into a weed. The example of rice domestication and de‐domestication indicates that endoferal traits can be attributed to human activity, such as changes in sowing (releasing human selection on shoot elongation) and threshing practices (releasing human selection on suppressed seed shattering). However, it might be more fruitful to look at the crop rather than at the weed: domestication creates selective pressures that are antagonistic to natural selection. This is most evident for seed dispersal—a seed that remains on the ear is lost for a natural or a weedy lifestyle; a seed that falls from the ear is lost for a domesticated lifestyle. Other traits are comparatively less channeled—a red pericarp per se is not a sufficient condition for a weedy lifestyle, however due to the antimicrobial effect of proanthocyanidins, it can turn into a necessary condition for weedy behavior at the very moment when a seed is shed. For culm length, the relation with weedy behavior is even more indirect.

Thus, in understanding ferality, it might be fruitful to consider functional interactions between different “weedy” gene loci (which are, by definition, also “domestication” loci). A wild (exoferal) allele for *Rc* can be perfectly consistent with domestication in combination with a nonfunctional allele for *SHA1/ SH4*, but the same allele, re‐combined with a re‐functionalized H_1_ allele for *SHA1/ SH4* will turn into a driver for evolution of a weed. Thus, some of the potentially weedy alleles (as seen for haplotype H_1_ of the *sd1* locus, or the *rufipogon* allele of the *Rc* locus) did not interfere with human use in the Indian landraces, because they were not linked with seed shed. In the very moment that the same alleles were recombined with shattering, they unfolded their weedy potential. Thus, “weedy genes” might not exist, and ferality might be rather a holistic trait that is *emerging* from recombination of hitherto unlinked alleles. If this holistic trait is translated into the realm of molecular biology, it should be reflected as interaction—either on the functional level (when two mutations act at different positions of a developmental pathway), or even on the level of direct protein–protein interaction.

Thus, to contain ferality it is not sufficient to select against individual alleles in one locus, but it is essential to detect and disrupt *combinations* of potentially weedy alleles of several loci.

## CONFLICT OF INTEREST

None declared.

## AUTHOR CONTRIBUTIONS

**Annabelle Grimm:** Investigation (lead); writing–original draft (lead). **Vaidurya P. Sahi:** Data curation (supporting); investigation (supporting); supervision (supporting); writing–review and editing (supporting). **Manuel Amann:** Data curation (supporting); investigation (supporting). **Francesco Vidotto:** Data curation (supporting); investigation (supporting). **Silvia Fogliatto:** Data curation (supporting); investigation (supporting); methodology (supporting); resources (supporting). **Katrien M. Devos:** Conceptualization (supporting); data curation (supporting); methodology (equal); supervision (equal); writing–review and editing (supporting). **Aldo Ferrero:** conceptualization (equal); investigation (supporting); supervision (supporting). **Peter Nick:** Conceptualization (equal); funding acquisition (lead); supervision (lead); visualization (equal); writing–original draft (equal); writing–review and editing (equal).

## Supporting information

Data S1

Data S2

Data S3

Table S1

Table S2

Table S3

Supplementary Material

## Data Availability

The data that support the findings will be available in the data sharing repository DRYAD. As per the requirement of the journal, the data will be made available as soon as the MS is accepted (https://datadryad.org/docs/JournalLookup.pdf).
