# Supplementary material for: Italian weedy rice—A case of de‐domestication?
Source: Ecol Evol. 2020 Jul 12;10(15):8449–64. doi: 10.1002/ece3.6551 (PMC7417233; doi:10.1002/ece3.6551)
Supplement: Supplementary file 7 — Supplementary Material [file ECE3-10-8449-s007.docx]

Accessions used in this study

| **Cultivar Name** | **Cultivated since (if available)** | **Weedy Rice Accession No.^*^** | **Collection Zone^*^** | **Weedy Rice Accession No.*** | **Collection Zone^*^** |
| --- | --- | --- | --- | --- | --- |
| Arborio | 1946 | TO1 | 4 | TO73 | 2 |
| Artiglio | ? | TO5 | 7 | TO75 | 4 |
| Baldo | 1964 | TO11 | 7 | TO79 | 8 |
| Ballila | 1924 | TO13 | 7 | TO81 | 8 |
| Bertone | 1829 (no longer grown) | TO19 | 7 | TO87 | 8 |
| Carnaroli | 1945 | TO20 | 3 | TO89 | 8 |
| Centauro | ? | TO24 | 3 | TO90 | 8 |
| Clearfield (BASF) | ? | TO26 | 3 | TO94 | 9 |
| Creso | ? | TO30 | 3 | TO101 | 9 |
| Flipper | 1997 | TO32 | 1 | TO106 | 9 |
| Gladio | ? | TO38 | 1 | TO108 | 9 |
| Lady Wright | ? (no longer grown) | TO41 | 2 | TO113 | 9 |
| Loto | 1988 | TO43 | 1 | TO116 | 8 |
| Originario Cinese | ? | TO46 | 2 | TO122 | 10 |
| Ostiglia | 1850 (no longer grown) | TO52 | 2 | TO126 | 10 |
| Prometeo | ? | TO54 | 2 | TO133 | 10 |
| Ranghino | 1887 (no longer grown) | TO57 | 4 | TO135 | 10 |
| Selenio | ? | TO60 | 5 | TO140 | 10 |
| Thaibonnet | ? | TO64 | 5 | TO143 | 10 |
| Vialone Nano | 1937 | TO67 | 5 | TO146 | 10 |

^*^ further information on the accessions and collection zones is available in Grimm et al. 2013

Primer Sequences

| **Name** | **5'-3' sequence** | **%GC** | **T_m_** | **Size** | **Reference** |
| --- | --- | --- | --- | --- | --- |
| qSH1_1_fw | ACCGGCAGTACTACCAGCAG | 60 | 60.33 |  | designed using Primer3 |
| qSH1_1_rev | CTGATGAzTGCACGCTATGCT | 50 | 60 | 1790 bp | designed using Primer3 |
| qSH1_2_fw | CACGCAACCAGGTAAATAGAAA | 40.91 | 59.18 |  | designed using Primer3 |
| qSH1_2_rev | GCTAAGCCCATTTCGTCATC | 50 | 59.67 | 1008 bp | designed using Primer3 |
| rc_015_for | CTGAAGGAAGTGATGACAACAAGACC | 46 | 67 |  | Gross et al. (2010) |
| rc_015.2_rev | TTAAGTATGACTTATATTTTACATATTTGCAC | 21 | 59 | 570 bp | Gross et al. (2010) |
| SD1_1_fw | CAACACAGCGCTCACTTCTC | 55 | 59.78 |  | designed using Primer3 |
| SD1_1_rev | AATCACGTCAGGTCGGTTTC | 50 | 59.97 | 1181 bp | designed using Primer3 |
| SD1_2_fw | GGGAATTGTTGTGTGTGCAG | 50 | 60.01 |  | designed using Primer3 |
| SD1_2_rev | GTACAGCGGTAGGGTCCAAA | 55 | 59.99 | 468 bp | designed using Primer3 |
| SDR4_fw | GCCTTCTTAACCCCACCAC | 57.98 | 59.4 |  | designed using Primer3 |
| SDR4_rev | TTAGAACCTGGCCTTGCATC | 50 | 60.21 | 1276 bp | designed using Primer3 |
| SH4_1_fw | CGCTCGGTTGATTAGGAGAG | 55 | 59.97 |  | designed using Primer3 |
| SH4_1_rev | CACACTGCACGCAGCTTTAT | 50 | 60.08 | 1357 bp | designed using Primer3 |
| SH4_2_fw | ATTGCGAAATCACTCGCTTT | 40 | 59.85 |  | designed using Primer3 |
| SH4_2_rev | TGCAGCCATTCCAAACAATA | 40 | 60.07 | 861 bp | designed using Primer3 |
| SHAT1_fw | TTGCAGATGAGCAACCTGAC | 50 | 59.99 |  | designed using Primer3 |
| SHAT1_rev | GATGAATGCAGCGATCTTGA | 45 | 59.91 | 1449 bp | designed using Primer3 |
| VP1_1_fw | ATAAGTGGGCCCAGAGGAAA | 50 | 60.82 |  | designed using Primer3 |
| VP1_1_rev | TCTTCTGGAGGTGGTGGTTC | 55 | 60.09 | 1070 bp | designed using Primer3 |
| VP1_2_fw | CTCACGAGCAACCGTGAGTA | 55 | 60.05 |  | designed using Primer3 |
| VP1_2_rev | GCTCTGCTTCAGCACCTTCT | 55 | 59.9 | 1251 bp | designed using Primer3 |
| VP1_3_fw | GCATGCAGACGATTGACATC | 50 | 60.24 |  | designed using Primer3 |
| VP1_3_rev | TAGCGCTACGATTCACATGC | 50 | 60.01 | 1961 bp | designed using Primer3 |
